# Nativity Homogeneity in Social Networks and Prostitution Patronage Among Male Migrant Laborers

**DOI:** 10.1007/s10461-018-2357-1

**Published:** 2018-12-11

**Authors:** Xiaozhao Yousef Yang, Tingzhong Yang

**Affiliations:** 1 Department of Sociology and Social Work, Sun Yat-sen University, Guangzhou, China; 2 Department of Political Science and Sociology, Murray State University, Murray, KY USA; 3 Center for Tobacco Control Research, Zhejiang University, Hangzhou, China; 4 Children’s Hospital, Zhejiang University School of Medicine, Hangzhou, China

**Keywords:** Prostitution patronage, Social network homogeneity, Nativity, Mediation analysis, Migration

## Abstract

Previous studies have repeatedly found the association between network homogeneity based on native-place and sexual risk behaviors among migrants. However, it remains unclear why such a simple numerical property of network composition can be correlated with a sexual risk behavior. Using a dataset (n = 1591) with detailed information on the sexual behaviors among male migrant laborers in the two Chinese provinces with the highest migrant inflows, we confirmed network homogeneity is significantly associated with prostitution patronage. With structural equation modeling, we further found that half of network homogeneity’s impact on prostitution patronage is mediated by three factors: peer deviance, family bonds, and hedonistic subcultural beliefs. In addition, network homogeneity’s association with hedonistic subcultural beliefs is fully mediated by peer deviance. Although the nativity homogeneity in social networks is still associated with prostitution patronage, more proximate psycho-social factors are found responsible for the network effect. Health policies seeking to integrate migrant laborers, removing the policy barriers against family bonds, and providing alternative sources of social support are highly promising for reducing sexual risk behavior among this population.

## Introduction

Commercial sex and unprotected commercial sex create a considerable risk in the contraction of STDs/HIV among migrant laborers around the world [1–4]. Many have called for attention to the potential outbreak of STDs/HIV circulating between sex workers, male migrant laborers, and the migrants’ families [5–7].

There are rich discussions on the individual attributes of prostitution patrons, mostly focusing on socioeconomic status and psychological correlates. However, both migration and prostitution patronage are not just an individual choice but a network-based chain of actions and subject to interpersonal influence. Studies on individual attributes do not answer how prostitution patronage is enabled by social structures, particularly how transactional sex as a choice in the “mate market” is patterned by the pool of associates that one routinely interacts with [8]. In response, the composition of social networks has recently attracted some long due attention.

Social networks refer to “relationships among social entities, and [focus] on the patterns of these relationships” [9]. The network approach has been employed in numerous studies in order to understand a range of at-risk health behaviors (for a review see Reference [10]. Regarding sexual risk behavior, social network analysis explains how they are constrained and channeled by “roles, positions, and local brokers”, and it emphasizes the actual composition of a pool of potential sexual partners [8]. In this study, we attend to a particularly important feature of a social network among migrants—network homogeneity by origin-place, and investigate the mechanism that network homogeneity affects prostitution patronage.

## Literature Review

### Social Network Homogeneity

Among different properties of a social network, network homogeneity is a particularly important concept for understanding migrants’ social and sexual behaviors. Network homogeneity describes the extent to which network alters are similar in terms of some chosen standards, such as gender, belief, race, origin-place, etc. Network homogeneity shapes peer pressure, information and resource diffusion, subcultural beliefs, and cohesiveness for individuals embedded in the homogenous networks [9, 11–13]. For migrant populations in particular, the characterizing feature of their social networks is that a large portion of their associates are other migrants coming from the same hometown or origin place, thus forming highly homogenous social networks in terms of origin-place [14, 15].

Although some studies suggested that homogenous networks may prevent sexual risk behaviors by reducing loneliness and providing migrants with more social support [16, 17], others have so far preponderantly found that homogenous networks are associated with a variety of at-risk behaviors. Homogenous networks consisting of the same native-place friends contribute to higher likelihood of buying sex and unprotected sex among Chinese rural migrants [18]. Compared to a simulated random network, networks laden with at-risk sexual behavior and needle-sharing show greater cohesiveness and transitivity, which conceptually and arithmetically correlate with network homogeneity [19]. Having homogenous networks or living in a homogenous neighborhood constitute a risk factor for unprotected sex and drug use, due to a stronger deviant subculture and peer pressure [20, 21]. Other than sexual risk behaviors, scholars also found that immigrants whose social networks are composed of homogeneous ties are more likely to use substances [22–24] and suffer from psychological adversity [14, 25].

### The Mechanism that Homogenous Networks Affect Prostitution Patronage

While sexual risk behaviors are found more prevalent among migrants with homogenous networks, the specific mechanisms at work to link network homogeneity and prostitution patronage have not been clearly specified and tested. To better understand the network pattern of prostitution patronage, it is crucial to consider the mechanisms through which network homogeneity exerts its influence. This missing link is again important because policy interventions require an understanding of the causal mechanisms behind correlated phenomena. Laumann and others, as an exception, argue that the local contextual factors including social networks shape an individual’s sex choice through cultural norms, the availability of desirable mates, and contact opportunities [8]. Based on the current literature, we argue that network homogeneity in terms of origin-place may induce prostitution patronage among migrants through three focal factors: peer deviance, social bonds with family, and hedonistic subcultural beliefs.

#### Peer Deviance

Since prostitution is an illegal or underground activity in most countries [26], a novice’s success in patronizing a sex worker without any knowledgeable peers is severely limited. Having knowledgeable peers who engaged in sexual risk behaviors significantly increases one’s own likelihood of following suit [27–29]. Deviant peers are necessary for initiating prostitution patronage, and they often deliver the required skills and scripts for a successful commercial sex transaction [30, 31].

Network homogeneity may be associated with peer deviance among migrants. While socioeconomically advantaged individuals can afford or even benefit from having homophilous ties to other advantaged individuals [32, 33], homogenous networks composed of similarly marginalized migrant peers may breed sexual risk behaviors. The “strength of weak ties” theory found that homogenous ties are ineffective in transmitting diverse information and resources that may connect individuals across the multiplexity of social settings [34]. The predominance of homogenous ties in migrants’ social networks also prevents sufficient interaction with the higher social strata, creating a barrier against assimilation and exchange [35, 36]. Alternatively, individuals connected to diverse types of social groups can better control the flow of resources and information, acting as brokers in their networks [37, 38]. Therefore, a homogenous network often becomes a synonym for being trapped in a poor and segregated network. Cloward and Ohlin [39] outlined the possibility that deviance arises out of the differential access to heterogenous sources of the means of satisfaction, where homogenous networks among the underprivileged present abundant deviant means of satisfaction. Due to the structural strain [40], a homogeneous network features more peers who resort to deviance in the face of unsurmountable stratification. Recently, Browning and colleagues [41] found that communities with dense homogenous ties become tolerant of deviance. They implied that homogenous networks are facilitative to peer deviance because people are more likely to acquiesce to the delinquencies conducted by others similar to themselves. Haynie [42] showed that the deviance-peer association is stronger in cohesive networks. Two studies in different sites showed how trans-local ties to heterogeneous groups moderated migrants’ experience of victimization [14, 25].

**Fig. 1:**
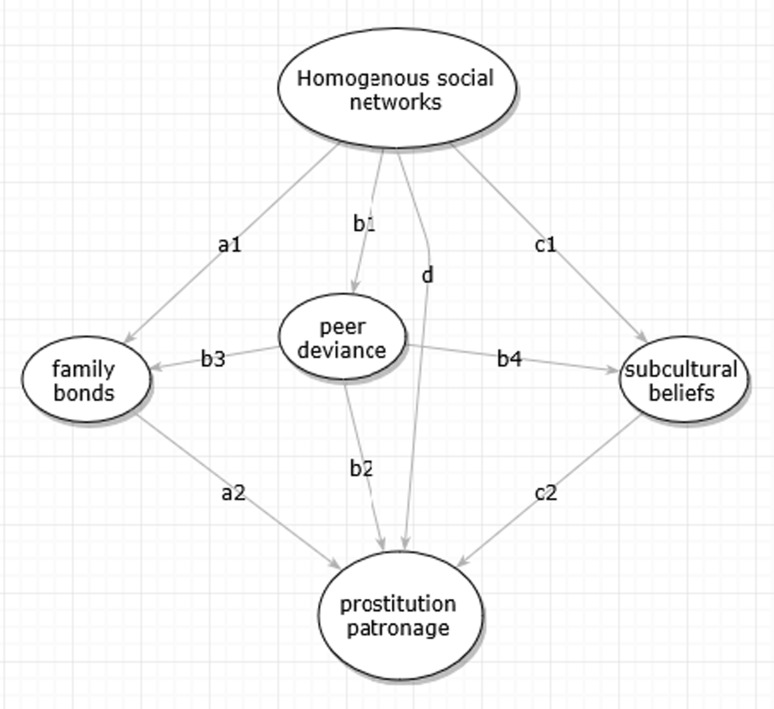
Conceptual pathways of the proposed model

Taken together, we hypothesize *H1: peer deviance is associated with network homogeneity. It mediates the association between network homogeneity and prostitution patronage (path b1–b2 in Fig.* 1).

#### Social Bonds with Family

Social bonds with the conventional institution of family is an important inhibitor of sexual risk behaviors [43, 44]. For migrants, the strength and quality of family bonds may depend on their network homogeneity by origin-place. The general law of structural hole dictates that the opportunity to know different people and acquire quality information is greater for the occupants of a position connecting heterogenous populations [37]. Network homogeneity reduces the flow of useful information and resources and creates a redundancy of social ties, while social status and prestige are conferred by one’s social connections [45]. Thus, homogenous social networks may place migrants in an unfavorable position of establishing marriage and a family. It was found that African American women are much less likely to get married because their social networks contain very few bridging connections across communities [8]; Chinese migrant laborers with homogenous social networks are confined to a small social circle that restricts social status and provides few opportunities for socializing across networks [46]. Furthermore, unlike non-mobile individuals who may nevertheless find marriage partners from a homogenous social unit such as church, college class, or social club, migrant laborers’ homogenous networks are often transitory and composed of same-gender peers [7, 15]. Therefore, migrants with more homogenous networks tend to acquaint fewer potential partners suitable for marriage and are less likely to have stable families. As a result, they see an elevated risk of using commercial sex.

Taken together, we hypothesize that *H2: Family bonds are weaker in homogenous networks. Family bonds mediate the association between network homogeneity and prostitution patronage (path a1–2 in Fig.* 1).

#### Subcultural Beliefs

Subcultural beliefs refer to a set of values and attitudes that violate or rebel against the mainstream norms of behaviors. Compared to actual peer deviance, subcultural beliefs are normative definitions held by individuals, and may be enforced by deviant peers. Given its relative definition to the mainstream norms, there are multitude types of subcultural beliefs depending on the specific nature of the concerned behavior. For sexual risk behaviors particularly, hedonistic subcultural beliefs prove to be a close associate with the actual conduct of a sexual risk behavior [47–49]. While the cultural institution of modern industrial societies promotes a rational and calculated behavioral mode to regulate sexual behaviors [50], hedonistic subcultural beliefs value immediate satisfaction over the delayed gratification achieved through institutionally sanctioned means. Hedonistic subcultural beliefs also endorse risk-taking as an essence of masculinity and emphasize the value of pleasure-seeking and corporeal satisfaction [51, 52].

Subculture beliefs can originate from prolonged and patterned interactions within a small circle of individuals [53]. Homogenous social networks provide just such a fertile ground for the emergence and reinforcement of subcultural beliefs. It is found that homogenous networks are facilitative for the formation and transmission of non-conforming attitudes [54]. Among migrant laborers, alternative criteria of confirming one’s social status and self-esteem will be established to compensate for one’s lack of recognition by the mainstream society. Cohen described the consequence of such social condition as “one solution is for individuals who share such problems to gravitate towards one another and jointly to establish new norms, new criteria of status” [55]. The sense of frustration and segregation resulted from homogenous networks then leads to the pursuit of hedonism, consumption, and immediate satisfaction [51]. Previous studies have observed among many insular and homogenous networks the hedonistic subcultural beliefs that prioritize hypermasculinity, risk-taking, pleasure-seeking, and conspicuous consumption [56–58]. Taken together, we hypothesize *H3: There is a higher level of hedonistic subcultural beliefs in more homogenous networks. Hedonistic subcultural beliefs mediate network homogeneity and prostitution patronage (path c1–c2 in Fig.* 1).

### Relationships Between the Mediators

Peer deviance, family bonds, and subcultural beliefs may be closely correlated, which requires us to consider their confounding relationships. Peer deviance may be directly associated with weaker family bonds, so that network homogeneity is indirectly associated with family bonds through peer deviance. Some studies emphasized peers as a source of deviance learning in competition with families and schools [59, 60]. One such study showed the non-significance of parents and schools in adolescent delinquency once peer effects were controlled for [61]. Thus, we hypothesize *H4a: peer deviance mediates the association between network homogeneity and family bonds (path b1–b3 in Fig.* 1). Network homogeneity may also indirectly influence subcultural beliefs through peer deviance, because deviant peers pose the potential to change one’s own normative definition about a deviant behavior and lead him to perceive the deviance as favorable [62]. Having more friends engaging in deviant activities may change the normative appeal of such activities after repeated exposure (see [63, 64]. This leads to hypothesis *H4b: peer deviance also mediates the association between network homogeneity and subcultural beliefs (path b1–b4 in Fig.* 1).

## Methodology

### Data

Among all, perhaps the most pressing need for understanding the mechanism of prostitution patronage among migrants comes from the case of China, whose nearly 200 million migrant laborers today make the largest mobile population in human history [65]. Compared to non-migrants in China, migrant laborers have a considerably elevated risk of using commercial sex (10–27% vs. 6–10%) [1, 66, 67]. This study’s data source, the China Migrant Sexual Health Survey, used a multi-stage systematic sampling procedure to recruit male migrant laborers in two Chinese cities in 2011. Two cities (Hangzhou and Canton) were selected for good reasons: both are hubs of migration influx in coastal China and are the capital cities of the provinces that received more than 30% of China’s migrant laborers [68].

Male migrant laborers were identified and selected through multistage sampling and were contacted at their work sites or dormitories by medical professionals from local health units, who had received a short protocol training prior to the survey. First, two districts were randomly selected from each city. Worksites were then used as the sampling units. Six categories of worksites were used to help insure diversity among participants: (1) construction, (2) machinery and transportation, (3) spin electronics, (4) family services, (5) business, and (6) others. Then, a quota-sampling procedure was used to recruit a composite sample approximately proportionate to the overall distribution of the migrant population by occupation clusters. Finally, we confirmed that selected participants were rural–urban migrant workers who were male, aged 18 years or older, held rural hukou (that is, were registered residents in a rural area), and had resided in a destination city for at least 6 months. The survey was self-administered individually in residences or in a secluded area away from colleagues, but staffs were present to guide the question flow and assist those with literacy difficulty. Response rate at the final stage was 84.4%. Respondents were given a small token of appreciation (toothbrush and tooth-paste) and a health education brochure for their participation in the study. The survey protocols were approved by the IRB at the correspondence author’s institution (#03BSH010).

### Measurement

For prostitute patronage, the survey started with a filter question “have you had non-marital sex.” Respondents then moved to another question “if yes, what type of people did you have non-marital sex with: fiancée, mistress, girlfriend, prostitute, others”—more than one type of sex partner could be indicated. Those who chose “prostitute” were again directed to another two questions: “how many times have you been to a prostitute” and “how many prostitutes have you had sex with”. The respondents answered these questions from two scales consisting of 1, 2–3, 4–5, 6–7, 8–9, more than 10 times/people. Because these two count variables may heavily depend on the length of stay in the city, it is essential to adjust for the time exposure factor. We summated these two variables for Poisson regressions and use the length of stay in the city as an offset. For Structural Equation Modeling, we log-transformed the raw variable plus 1 and divided the log product by length of stay in the city to arrive at a time-adjusted prostitution patronage indicator.^1^ The length of stay in the city is the number of months per year filled in by the respondents.

We measured peer deviance by self-reported percentage of deviant peers. The survey asked respondents to estimate the percentage of their friends who had received massages from a girl in beauty salon and bath center, had sex service in an entertainment venue, had sex with a prostitute in general.

For family bonds, we adopted indicators “where is your wife when you work away from your hometown” and “where are your children when you work away from your hometown”. Those who answered ‘stays with me’ were coded 1 to indicate the existence of a family bond. Months spent with family every year is another indicator of the strength of family bonds.

We measure hedonistic subcultural beliefs by a set of questions assessing the beliefs about immediate satisfaction and hedonistic pleasure-seeking because strong beliefs in sensation- and pleasure-seeking have proven to be correlated with prostitution patronage [48, 49, 69], and other sexual risk behaviors [47]. On seven-point Likert scales, respondents rated their level of agreement for pleasure-seeking statements: “life is a dream, its purpose is to enjoy pleasure”, “money should be first spent on having fun”, “you can never worry about enjoying too much pleasure”, “time flies, you should have fun as soon as possible^2^”. The scale has been established and used in previous studies [18, 70, 71].

Other covariates include income, education, age, length of stay in the city, health behaviors (smoking and drinking), and health knowledge about HIV.

### Statistical Analysis

The classic approach to mediation analysis is a three-step procedure proposed by Baron and Kenny [72]. Basically, if the association between independent and dependent variables differs in magnitude or in significance after a mediator is introduced, one could conclude with mediation effect. We followed this classic approach by presenting step-wise Poisson regressions for count data, and examined whether the coefficient of network homogeneity is reduced after peer deviance, family bonds, and subcultural beliefs entered the model.

However, recent scholarship has pointed out some issues with the classic approach and called for a formal test on mediation effect [73–75]. As a supplement, we also used Structural Equation Modeling (SEM) to formally test the proposed mediatory relationships. SEM provides a comprehensive comparison of direct effects, indirect effects, and total effects for mediation analyses. SEM not only calculates direct effects, indirect effects, total effects, and the ratio between them, it also tests the significance of these parameters, thus informing us of the magnitude and significance of the mediations. When combined with bootstrap resampling, SEM quantifies mediation effects with confidence intervals, which is a recommended approach for mediation analysis [76]. Therefore, SEM is acclaimed as a supplement or replacement to the classic mediation analysis based in comparing the changes in regression parameters [73–75].

We used the weighted least square with mean–variance with robust errors for estimating the SEMs and performed 277 bootstrap resampling for Confidence Intervals. We first confirmed the validity of the measures using confirmatory factor analysis. Then, we presented three different SEMs with different mediators. We tested mediation effects by comparing the changes in statistical associations after mediators were introduced, and also by the bootstrapped ratio of direct effect to total effect. The For the goodness of fit of SEMs, we reported fit indices and their versions after robust error adjustment [77]: the Tucker-Lewis Index (TLI), Comparative Fit Index (CFI), and the root mean square error of approximation (RMSEA). CFI and TLF are considered satisfactory when above 0.9, and are excellent when above 0.95. RMSEA below 0.08 is satisfactory, it indicates excellent fit when below 0.05 [78]. The SEMs were estimated with R’s lavaan package [79].

## Results

Table 1 shows the descriptive statistics of the sample. A median male migrant laborer had solicited approximately two sex workers and had done twice. The median network homogeneity is 0.69, back transform it to exponential value gives us 0.99 (= exp(0.69)−1). This close-to-one ratio indicates that migrant laborers have nearly equal number of same-town friends and generic friends in their social networks.

**Table 1:** Descriptive statistics of the sample

N = 1583	Proportion (%)	Median (IQR)
Network homogeneity by nativity (log-transformed)		0.69 (0.29)
Times solicited sex worker (log-transformed and time adjusted)		0.69 (0.69)
Number of sex workers (log-transformed and time adjusted)		1.10 (1.26)
Pleasure-seeking beliefs		
Life is to enjoy pleasure		4.0 (3.0)
Money should be used for fun		4.0 (2.0)
Don’t worry about having pleasure		4.0 (2.0)
Having fun as soon as possible		4.0 (3.0)
Bonds with family		
Stay with wife	31.8	
Stay with child	17.4	
Months unite with family		1.0 (4.0)
Percentage of deviant peers	45.3	
Patronized beauty salons	48.5	
Been to sauna	44	
Been to leisure venues	44	
Solicited sex worker	48.5	
Length of stay in the city		7.0 (2.0)
Age (min = 16, max = 64)		30.0 (11.0)
Income (1 ≤ 1000CNY; 6 = 5000CNY+)		3.0 (3.0)
Education		
Elementary school or below	11.0	
Junior high	52.3	
High school or vocational	25.8	
College or above	10.9	
HIV safe belief		4.67 (2.0)
Smoking	64.6	
Drinking	46.3	

We used Poisson regression to model the relationship between network homogeneity and prostitution patronage. The first model in Table 2 included network homogeneity and control variables, and shows a clear and significant association between network homogeneity and prostitution patronage. For every one unit increase in network homogeneity, the migrants will patronize 0.26 more sex workers or 0.26 more times. Model 2 further introduced the three variables that are hypothesized as the mediators of prostitution patronage. The inclusion of family bonds, peer deviance, and subcultural beliefs reduces the magnitude of the effect of network homogeneity, but does not eliminate the latter’s statistical significance. These mediators are themselves significantly associated with prostitution patronage in a direction confirming the hypotheses. If we follow the classic procedure of establishing a mediatory relationship, we may conclude there is at least a partial mediation effect since the necessary conditions of the classic approach to mediation analysis were fulfilled.

**Table 2:** Poisson regressions on summated level of prostitution patronage

	Model 1 (n = 1515)		Model 2 (n = 1515)	
	Coefficient	SE	Coefficient	SE
Network homogeneity	0.26***	0.03	0.14***	0.03
Income	0.01	0.01	− 0.00	0.01
Education	0.07***	0.02	0.07***	0.02
Age	− 0.00	0.00	− 0.00	0.00
Smoking	0.32***	0.03	0.25***	0.03
Drinking	0.12***	0.03	0.18***	0.03
HIV knowledge	− 0.02**	0.01	0.02**	0.01
Length of stay	− 0.01	0.01	0.03***	0.01
Family bonds			− 0.01*	0.00
Peer deviance			0.01***	0.00
Subcultural belief			0.05***	0.01

*p < 0.05, **p < 0.01, ***p < 0.001

As discussed in the Methodology section, the classic approach to mediation analysis must be supplemented by more rigorous tests. Thus, we resort to SEM to test the potential mediatory relationships. Before proceeding to the SEM, we tested the internal reliability of the construct using Cronbach’s alpha and reported in Table 3. The three exogenous constructs all have satisfactory alpha, and they are significantly correlated except for the pair of family bonds and subcultural beliefs.

**Table 3:** Confirmatory factor analysis: covariances between latent variables and internal reliability (α) of latent variables

	Peer deviance	Family bonds	Subcultural beliefs
Peer deviance	626.07 (31.36)		
Family bonds	− 1.72 (0.03)	0.26 (0.02)	
Subcultural beliefs	12.45 (1.56)	− 0.01 (0.02)	2.46 (0.13)
Cronbach’s α	0.89	0.70	0.92
Goodness of fit	Χ^2^ (df = 59) = 190, CFI = 0.98, robust CFI = 0.90, TLI = 0.98, robust TFI = 0.87, RMSEA = 0.04, robust RMSEA = 0.06		

**Fig. 2:**
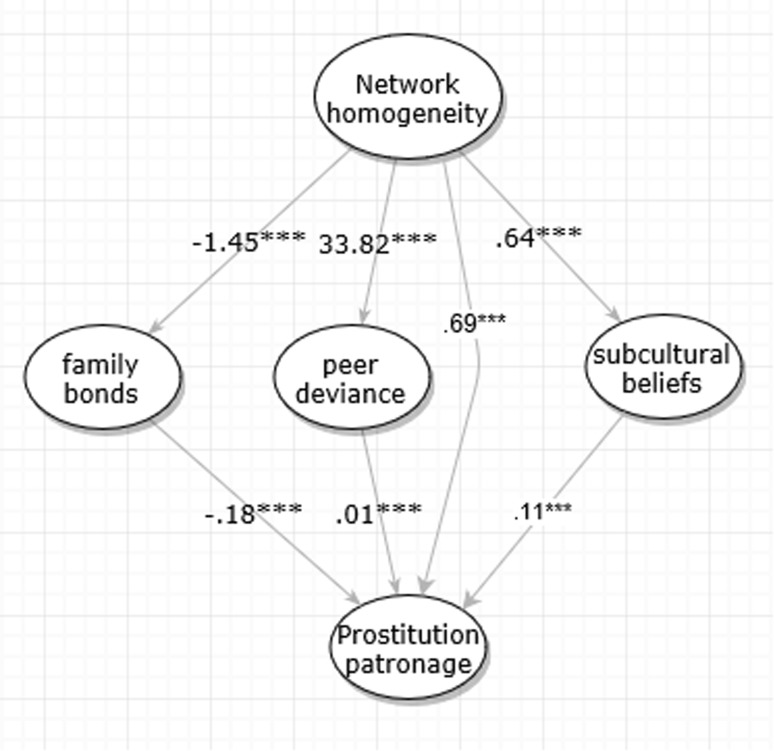
Mediation model for network homogeneity and prostitution patronage. CFI = 0.87, TLI = 0.93, RMSEA = 0.11

The mediatory effects are first tested and shown in Fig. 2. Here, network homogeneity’s effect is specified to go through the three mediators and also points directly to the dependent variable. With this specification, although network homogeneity is still significantly associated with prostitution patronage (0.69, p < 0.001), much of the association’s strength has been diverted to go through the mediators. The magnitude of aggregated indirect effects is 0.67 (33.82*0.01 + 1.45*0.18 + 0.64*0.11), almost the same amount as the direct effect. By this criteria, we may claim that half of the total influence that network homogeneity has on prostitution patronage is actually exercised through the mediators. Network homogeneity has a significant and negative association (− 1.45, p < 0.001) with family bonds, and family bonds itself is negatively associated with prostitution patronage (− 0.18, p < 0.001); network homogeneity is positively associated with peer deviance (0.40, p < 0.001), and peer deviance is also positively associated with prostitution patronage (33.82, p < 0.001), and it increases subcultural beliefs (0.64, p < 0.001), which in turn increases prostitution patronage (0.23, p < 0.001). Unlike the Poisson regressions that could not simultaneously test the relative contributions of structural variables, the SEM in Fig. 2 suggests a significant partial mediation effect. Hypotheses H1, H2, and H3 are all supported.

**Fig. 3:**
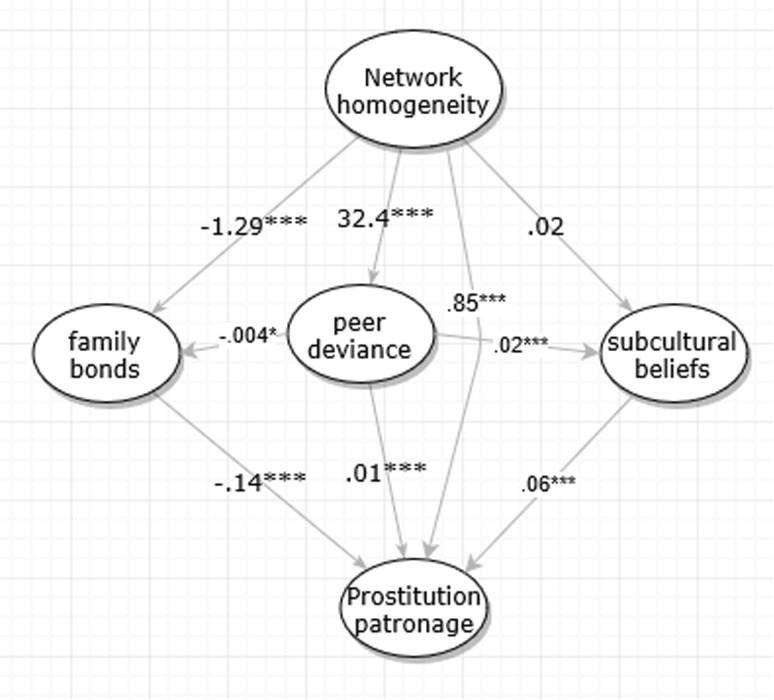
Mediation model for network homogeneity, peer deviance, and prostitution patronage. CFI = 0.99, TLI = 0.99, RMSEA = 0.057

SEM in Fig. 3 also suggests that, while network homogeneity is significantly associated with prostitution patronage (0.85, p < 0.001), the effect is mediated by the mediators we introduced. To quantify mediatory effects, we turn to Table 4 with robust and bootstrap-based estimates of the ratio of direct effect to total effect. In the lower panel of Table 4, the direct effect of network homogeneity on prostitution patronage is 0.85, with a bootstrapped Confidence Interval between 0.74 and 0.98. The ratio of direct effect to total effect is 0.58 with a bootstrapped Confidence Interval between 0.52 and 0.64. By this result, we are confident that the mediators explain away almost a half of network homogeneity’s effect on prostitution patronage.

**Table 4:** Unstandardized parameters of the final structural equation model, estimated with weighted least squares and mean–variance (WLSMV)

N = 1583	Exogenous variable			
Endogenous variable	Network homogeneity	Peer deviance	Family bonds	Subcultural beliefs
Peer deviance	32.40*** (2.84)			
Family bonds	− 1.29*** (0.15)	− 0.004* (0.002)		
Subcultural beliefs	0.02 (0.16)	0.02*** (0.002)		
Prostitution patronage	0.85*** (0.06)	0.01*** (0.001)	− 0.14*** (0.02)	0.06*** (0.01)

Network homogeneity	Direct effect	Indirect effect	Direct-total effect ratio	
Mediation parameters with robust errors (SE) and bootstrap resampling [95% C.I.]				
Family bonds	− 1.29 (0.15) [− 1.60, − 0.99]	− 0.14(0.06) [− 0.27, 0.00]	0.90 (0.04) [0.81, 1.00]	
Subcultural beliefs	0.02 (0.16) [− 0.33, 0.29]	0.65 (0.08) [0.49, 0.80]	0.03 (0.26) [− 0.69, 0.77]	
Prostitution patronage	0.85 (0.06) [0.74, 0.98]	0.62 (0.05) [0.52, 0.71]	0.58 (0.03) [0.52, 0.64]	
Goodness of fit	Χ^2^ (df = 69) = 345, CFI = 0.97, robust CFI = 0.91, TLI = 0.96, robust TFI = 0.89, RMSEA = 0.05, robust RMSEA = 0.06			

Fit statistics based on mean- and variance-adjusted Chi squares are at bottom

*p < 0.05, **p < 0.01, ***p < 0.001

Hypotheses H4a and H4b state that peer deviance mediates network homogeneity’s effects on family bonds and subcultural beliefs. Figure 3 shows that network homogeneity’s association with subcultural beliefs has been fully mediated by peer delinquency, suggesting that network homogeneity may not be able to directly change one’s normative beliefs. The same finding can be found in Table 4’s unstandardized coefficients, where network homogeneity is now unassociated with subcultural beliefs (0.02, p > 0.05). The ratio of direct effect to total effect found in Table 4 is a statistically insignificant 0.03 (95% C.I. between − 0.69 and 0.77). H4b is thus supported: peer deviance mediates the relationship between network homogeneity and subcultural beliefs. However, H4a has very weak support: family bonds are still significantly associated with network homogeneity (beta = − 1.29, p < 0.001); the ratio of direct effect to total effect between family bonds and network homogeneity is 0.90 (95% C.I. between 0.81 and 1.00). Network homogeneity’s effect on family bonds is almost unmediated by peer deviance. We conclude that H4b can be confirmed, while H4a is not supported. This last SEM has dramatically improved fitness indices. CFI, TLI, RMSEA, and their robust versions all fall into a satisfactory or excellent range.

## Discussion

In recent years, scholars have a renewed attention to the social network factors in sexual risk behaviors [10, 80]. One line of research in this subject has demonstrated that homogenous social networks are associated with deviant behaviors including prostitution patronage [18, 20, 21, 24, 42, 81, 82]. However, it has so far remained unclear why and exactly how network homogeneity is correlated with prostitution patronage. The current study hypothesized and tested that (1) network homogeneity is significantly associated with prostitution patronage; (2) this association is mediated by peer deviance, family bonds, and subcultural beliefs (H1, H2, H3); (3) network homogeneity also indirectly affect family bonds and subcultural beliefs through peer deviance (H4a, H4b). The results have advanced our knowledge in the mechanism of how social network homogeneity is associated with prostitution patronage.

This study’s findings support the hypothesis that network homogeneity’s effect on prostitution patronage is mediated by peer deviance, family bonds, and subcultural beliefs (H1, H2, H3). Network homogeneity is an important correlate of prostitution patronage, but half of its effect on prostitution patronage actually applies through the mediators.

The findings of this study point back to the behavioral consequences of network homogeneity and its importance in the formation of sexual risk behaviors. While having a certain degree of network homogeneity is nearly a default mode in people’s life [13, 83], scholars have shown that a homogenous network circulates redundant information and resources [34, 84, 85], while heterogenous networks are shown to facilitate upward mobility and resource acquisition [37, 86–88]. This phenomenon is particularly amplified in marginal populations such as migrant laborers, whose primary socialization platform is homogenously composed of other migrants from their own hometowns. This is particularly relevant to the context of China, the traditional culture rooted in a collectivist management of kinships prioritizes hierarchal socialization. Native-place relationships are regarded as the most important non-kin social institution for people to build up support and resources. Following this vein, we demonstrated that a homogenous network is associated with prostitution patronage by breeding hedonistic subcultural beliefs, presenting more deviant peers, and weakening family bonds.

Our findings echo the tradition of formal sociology which emphasizes the equal importance of structures and individual psychosocial attributes in understanding sexual behaviors. Irrespective of individual attributes, social network structures shape the opportunity of forming friendship [88, 89], the speed of diffusing resources and information [84, 90], and the likelihood of deviance [21, 41, 42]. On the other hand, the overall structures do not exist in the vacuum, thus it was the intention of this study to answer why network homogeneity as a interpersonal structural force can be associated with prostitution patronage. A previous study discussed how the overall structure of sexual networks and individual sex norms together affected the chance of STD transmission [81]. Similarly in this study, network homogeneity provides a structuring channel to affect individual’s chance of marriage and forming a family, interacting with deviant peers, and developing deviant subcultural attitudes.

Finally, this study showed that peer deviance is associated with subcultural beliefs and mediates the link between network homogeneity and subcultural beliefs. Due to the cross-sectional nature of the study, we do not intend to answer whether peer deviance causally leads to subcultural beliefs, and it is possible that people with stronger subcultural beliefs will congregate in the deviant crowd. Instead, the results showed that network homogeneity is only indirectly responsible for the development of subcultural beliefs. On the other hand, network homogeneity is directly associated with weaker family bonds, pointing to the hypothesis that homogenous networks may have thwarted the male migrants’ success to form their own families, especially when such networks consist mostly of same-gender coworkers from the same origin-place.

Policy-wise, this study may inform policymakers of the practical interventions to reduce HIV/STDs infected through sexual risk behaviors. Prostitution patronage is a major risk factor in the transmission of HIV/STD in most countries where sex work is not properly regulated. By establishing the mediations between network homogeneity and prostitution patronage, this study advises policymakers to attend to the important mediatory factors leading to this sexual risk behavior. Informal associations such as homogenous friendship networks are often impossible to change by austere policies, therefore, policy makers should instead focus on more malleable factors that mediate the effects of network structure. They may seek to remove several arbitrary barriers preventing migrant laborers to establish family bonds, such as the household registration system and ID-associated security measures. Integration of migrants into the urban social life is necessary to reduce the prevalence of subcultural beliefs, including allowing the children of migrants to attend any public schools in their resident area and reducing the overall economic inequality. Similarly, it is also possible to weaken the influence of peer deviance by reducing migrants’ reliance on peer groups for resources and social support. Critical resources and information are often afforded to the marginal populace through organizations specialized in targeted resource distribution, and migrants receive the needed support of better qualify from these organizations [91]. If migrant laborers had any alternative support groups to rely on, such as labor unions, religious organizations, NGOs, they need not entirely conform their actions with peer group.

### A Note on Reverse Causality

The rich tradition in the deviance scholarship has brought to our attention the possible reverse causality and selection effect in our conceptual model. First, people who frequent sex workers may develop homogenous social networks as a consequence (reversing path d in Fig. 1). Second, peer deviance may be a projection of one’s own sexual risk behavior, rather than its antecedent (reversing path b2 in Fig. 1).

While the cross-sectional nature of this study does not allow a complete decomposition of the recursive pathways, concerns about reverse causality between the key variables can be ameliorated for two reasons. First, deviance’s selection effect is primarily discussed for peer selections, while network homogeneity by origin-place is unlikely a product of one’s own behavior. Homogenous networks and homophily are the default mode of human interactions due to the higher likelihood and greater cognitive comfort of socializing with similar others [12, 13]. For migrant laborers, having same-hometown friends is the default mode for leaving one’s hometown and starting new life in the cities [14, 15, 46]. This natural socialization platform is unlikely a result of individual choice.

Second, some argued that self-reported peer deviance may be irrelevant to the actual peer deviance and largely a projection of respondent’s own behavior [92, 93]. We dispute this with a cross-comparison in Table 5 in Appendix. The reported levels of peer deviance are consistently and significantly higher among those who initiated commercial sex in the company of peers or referred by peers, compared to those initiated on their own. Since both types of the respondents have conducted the same behavior, the variation in peer deviance cannot just be a projection of their own behavior but has to relate to the different *modes* of the behavior, i.e. whether peers were present at the time of buying sex. Therefore, our measurement of peer deviance indeed captured a significant variation in the actual peer behaviors.

Despite the various contributions this study provides, there are few points not addressed by this study. First, this is not a longitudinal study, mediations based on cross-sectional data rarely imply causation. Second, the survey did not incorporate vignette methods to reduce the potential social desirability bias regarding sexual behaviors. Third, family bonds are measured with binary indicators, future studies may design more meticulous questions on family bonds and the bonding strength.

## Appendix

See Table 5.

Methods of initiating commercial sex behavior were only asked for venue-specific sex behaviors. Therefore, there are only two types of respondents’ own commercial sex behavior for “sex with prostitute in general”: those patronized prostitutes and those who hadn’t.

**Table 5:** Estimated peer deviance by respondents’ own modes of initial commercial sex behaviors, with means and 95% confidence intervals (in the brackets) presented

Estimated percentage of friends who had used the following commercial sex service	“The first time you went there was”		
	Never patronized the place	“Discovered by chance and went alone”	“with friends”, or “after being told by friends”
Massage in beauty salon	32.46 (30.6, 34.4)	52.54 (48.9, 56.1)	60.7 (58.6, 62.7)***
	N = 622	N = 232	N = 746
Massage in bath center	27.5 (24.7, 30.3)	48.1 (44.5, 51.7)	61.5 (59.3, 63.7)***
	N = 412	N = 237	N = 624
Sex with prostitutes in entertainment venue	28.1 (25.1, 31.1)	44.3 (40.8, 47.8)	58.7 (56.7, 60.8)***
	N = 234	N = 202	N = 731
Sex with prostitute in general	26.9 (24.6, 29.2)	55.7 (54.1, 57.4)	
	N = 398	N = 1193	

***p < 0.001, two-sample Student’s *t* test with unequal variance comparing the estimated percentage of deviant peers between the “went alone” group and the peer-initiated group

## Footnotes

1 Let latent construct of prostitution patronage, L, be measured by times (t) and number (p) of prostitutes plus the random error, $L = \log \left( t \right) + \log \left( p \right) + \sigma. \log \left( {\frac{t}{expo}} \right) + \log \left( {\frac{p}{expo}} \right) = x + 2*\log \left( {expo} \right) + \sigma$, is functionally equivalent to $\log \left( {L|x} \right) = \log \left( {expo} \right) + \sigma$.

2 “Enjoy pleasure” is translated from Chinese *xiangle*, “have fun” is translated from *xingle*, both refer to actions that bring worldly and bodily pleasure..
